# Chagas disease-related mortality in Colombia from 1979 to 2018: temporal and spatial trends.

**DOI:** 10.1590/0037-8682-0768-2020

**Published:** 2021-02-26

**Authors:** Mario Javier Olivera, Julián Felipe Porras-Villamil, Juan Carlos Villar, Eliana Váquiro Herrera, Giancarlo Buitrago

**Affiliations:** 1 Instituto Nacional de Salud, Grupo de Parasitología, Bogotá D.C., Colombia.; 2 Universidad Nacional de Colombia, Facultad de Medicina, Bogotá D.C., Colombia.; 3 Fundación Cardioinfantil - Instituto de Cardiología, Departamento de Investigaciones, Bogotá D.C., Colombia.; 4 Universidad Nacional de Colombia, Facultad de Medicina, Instituto de Investigaciones Clínicas, Bogotá D.C., Colombia.; 5 Hospital Universitario Nacional de Colombia, Bogotá D.C., Colombia.

**Keywords:** Chagas disease, Mortality, Epidemiology, Time trends, Colombia

## Abstract

**INTRODUCTIOn:**

Studies on Chagas disease-related mortality assist in decision-making in health policies. We analyzed the epidemiological characteristics, temporal trends, and regional differences in Chagas disease-related mortality in Colombia from 1979 to 2018.

**METHODS:**

A time-series study was conducted using death records and population data from the National Administrative Department of Statistics, using categorizations from the International Classification of Disease (ICD)-9 and ICD-10 systems. All deaths with Chagas disease as an underlying or associated cause of death were included. Crude and age-sex standardized mortality rates per 100,000 inhabitants and the annual percent change (APC) were calculated.

**RESULTS:**

Of the 7,287,461 deaths recorded in Colombia during 1979-2018, 3,276 (0.04%) deaths were related to Chagas disease-2,827 (86.3%) as an underlying cause and 449 (13.7%) as an associated cause. The average annual age-sex standardized mortality rate was 0.211 (95% confidence interval [CI]: 0.170-0.252) deaths/100,000 inhabitants, with a significant upward trend (APC = 6.60%; 95% CI: 5.9-7.3). The highest Chagas disease-related death rates were in males (0.284 deaths/100,000 inhabitants), those ≥65 years old (1.296 deaths/100,000 inhabitants), and residents of the Orinoco region (1.809 deaths/100,000 inhabitants). There was a significant increase in mortality in the Orinoco (APC = 8.28%; 95% CI: 6.4-10.2), Caribbean (APC = 5.06%; 95% CI: 3.6-6.5), and Andean (APC = 4.63%; 95% CI: 3.9-5.3) regions.

**CONCLUSIONS:**

Chagas disease remains a major public health issue in Colombia with high mortality rates in older age groups, a wide geographic distribution, regional differences, and the potential to increase.

## INTRODUCTION

*Trypanosoma cruzi* infection is one of the most important autochthonous parasitic diseases in the Americas, causing disability and death^1^. The clinical course, progression, and survival of Chagas disease are highly variable and poorly understood^2^. The severity of this disease is associated with complex interactions among the genetic diversity of the parasite, host, and environmental factors^3^. In addition, barriers to accessing health services particularly affect its diagnosis and treatment^4^.

Chagas disease is considered a public health problem, with great economic and social repercussions^5^. Approximately 6-7 million people are infected worldwide, causing more than 7,000 deaths annually^1^. The disease has few treatment alternatives and high mortality rates that vary between countries and regions^6^ ^-^ ^8^. These differences in mortality rates could indicate changes in the effectiveness of control measures, transmission dynamics, *T. cruzi* genetic diversity, unequal recognition of the disease, and inequalities in care and diagnostic capabilities among other reasons^9^. 

In Colombia, with a significant reduction in vector and transfusion transmission, the number of new cases has decreased in recent years^10^. However, in the last decade, reports of acute cases of oral transmission have increased^11^. Recent studies have estimated that the prevalence of Chagas disease is approximately 2.0% (95% confidence interval (CI): 1.0-4.0) and is higher in the adult population at 3.0% (95% CI: 1.0-4.0)^12^. Few studies have been dedicated to identifying and describing changes in the analysis of the temporal and spatial trends of mortality due to infection by *T. cruzi* in Colombia.

Studying the trends of mortality rates assist in developing health policies, planning health services, and evaluating the impact of disease on the population^13^. In addition, they provide complete and accurate data, particularly about causes of mortality that guide high-quality decision-making in public health. Given the chronic nature of *T. cruzi* infection, increase in life expectancy in Colombia, and its high prevalence in the adult population, we can hypothesize that mortality rates of the Chagas disease will remain high in the coming decades. 

This study aimed to analyze the epidemiological characteristics, temporal trends, and regional differences in Chagas disease-related mortality in Colombia.

## METHODS

### Study setting

Colombia is located in the northwest of South America with an area of 1.141.748 km^2^ and a population of about 48.2 million^14^. It is characterized by geographical diversity due to its location and the presence of Andes Mountain range that spans from south to north. These conditions and their interactions with bioclimatic characteristics have delimited six natural regions of Colombia: (1) the Caribbean Region, located to the north along the coastal zone of the Atlantic Ocean; (2) the Pacific Region, located in the east along the coastal zone of the Pacific Ocean; (3) the Andean Region, situated in the center of Colombia, comprising the Andes Mountains and two inter-Andean valleys; (4) the Orinoco Region, located to the east of the Andes Mountains; (5) the Amazonia Region, also located to the east of the Andes Mountains; and (6) an insular region composed of islands, cays, and islets. Administratively, the country is divided into second-level territorial entities called departments (32) and districts (9) and third-level entities called municipalities (1,122)^15^.

### Study design

A time-series study based on secondary national mortality data, with an ecological perspective in regions of Colombia, was conducted using the Colombian National Death Registry database from the National Administrative Department of Statistics (DANE). We estimated the age-standardized mortality rates related to Chagas disease in a 40-year period from 1979 to 2018.

### Mortality and population data collection

The Colombian mortality information system has an open-source, online database, where all causes of death have been registered based on the death certificate in Colombia since 1979. We included all deaths that occurred in Colombia between 1979 and 2018. Anonymous data is freely available in the public domain on the website of DANE (https://www.dane.gov.co/index.php/estadisticas-por-tema/demografia-y-poblacion). Mortality and population data were retrieved from the DANE website. This database contains demographic characteristics, geographic location, and causes of death that have occurred in Colombia in recent decades. Primary, secondary, and contributing causes of death have been classified according to the 9th and 10th revisions of the International Classification of Diseases (ICD). Population estimations in Colombia were based on data from the National Population Censuses (2005).

### Definition of mortality associated with Chagas disease

Deaths related to Chagas disease were defined by the following ICD codes: 086 for the period 1979-1996 (ICD-9) and B57 for 1997-2018 (ICD-10). Causes of death for the B57 category comprised all subcategories (B57.0 to B57.5). Death due to Chagas disease was defined by the presence of any of the above ICD codes in any field of the cause of death-primary, secondary, or underlying causes of death.

### Data analysis

Descriptive statistics for the study population included the calculation of absolute numbers and proportions (along with 95% CI). Chagas disease-related mortality rates in Colombia were analyzed over a 40-year period from 1979 to 2018. Mortality and population data were organized into five-year age groups, up to 65+ years, to correspond with the age categories used in the DANE database and those provided in mortality and population data. Crude and age-sex standardized mortality rates, expressed per 100,000 inhabitants, were calculated for Colombia and its departments and geographic regions. The crude mortality rate was calculated as the ratio of the total number of deaths due to Chagas disease and the estimated population. The direct standardization method was applied to eliminate the effect of age-sex on mortality, using the Colombian population from the 2005 census as the standard. The age-sex standardized mortality rate was calculated as the ratio of the observed to expected mortality rate of patients and expressed per 100,000 inhabitants. Time trends were assessed by joinpoint regression analyses to determine the annual percent change (APC) in mortality rates to identify any significant change over time in a trend slope. Joinpoint regression analysis identifies the best fit for inflections at which there is a significant change in trend, using a series of permutation tests with Bonferroni correction for multiple testing. Each *p*-value corresponds to this type of test. In this study, joinpoint analysis was used to identify the independent variable of years at which significant changes in mortality rate occurred over the study period and the magnitude of these changes. Up to four joinpoints were allowed using a Monte Carlo permutation method, and we assessed whether there was a statistically significant difference from no change in each segment using a *p*-value <0.05. The statistical software R (R Foundation for Statistical Computing, Vienna, Austria; http://www.R-project.org/) and Stata version 14.0 (Stata Corporation LP, College Station, TX, USA) were used. Maps were created using QGIS v2.18.23 using publicly available shapefiles.

### Ethics

The Research Ethics and Methodology Committee of the National Institute of Health, Colombia, approved this study (registration number: CEMIN 17-2018).

## RESULTS

From January 1979 to December 2018, 7,287,461 deaths were registered in Colombia. In this period, 3,276 (0.04%) deaths had ICD codes related to Chagas disease, with 2,827 of them (86.3%) having an underlying cause and 449 (13.7%) having an associated cause of death. The mean number of deaths related to Chagas disease was 81.9 per year, ranging from 13 in 1979 to 222 in 2018. Over the four decades, the average crude mortality rate was 0.188 (95% CI: 0.14-0.23) deaths per 100,000 inhabitants, and the age-sex standardized mortality rate was 0.211 (95% CI: 0.170-0.252) deaths per 100,000 inhabitants.

### Epidemiological characteristics

Most individuals who had Chagas disease as their cause of death were male (62.5%), and the highest proportion of deaths (86.3%) occurred after 45 years of age, particularly among those aged ≥65 years (45.4%), followed by those aged 45-64 years (40.9%). The health insurance regime was available to 2,767 (84.5%) individuals who died due to Chagas disease. Most of the deaths occurred among people affiliated with the subsidized regime, i.e., people with incomes below the current legal monthly minimum wage (Table 1). Regarding coinfections, 2.9% of deaths related to Chagas disease were associated with the human immunodeficiency virus (HIV) infection, 56.8% of which were observed in young adults and middle-aged people (15-44 years old). Information on job occupation was available for only 43.2% of the registrants. Of those, 15.9% were farmers, 11.3% were housekeepers, and 16% were others. In terms of ethnicity, 27.6% of the cases occurred in the Afromestizo population. Most of the deaths occurred in hospitals (83.8%) and in urban areas (93.2%).

**TABLE 1:** Epidemiological aspects, number of deaths and age- and sex-standardized mortality rates (per 100,000 inhabitants) related to Chagas disease in Colombia from 1979 to 2018.

Epidemiological aspects	Deaths (n)	Deaths (%)	Mortality rates	95% CI
			(per 100,000)	
**Chagas disease-related deaths**	3,276	100	0.211	(0.170-0.252)
**Sex**				
Female	1,229	37.5	0.147	(0.116-0.179)
Male	2,047	62.5	0.284	(0.231-0.337)
**Age (years)**				
0-4	5	0.2	0.001	(0.000-0.003)
5-14	14	0.4	0.005	(0.001-0.008)
15-44	430	13.1	0.059	(0.052-0.065)
45-64	1,341	40.9	0.508	(0.429-0.585)
≥65	1,486	45.4	1.296	(0.938-1.641)
**Affiliation**				
Contributory	1,163	42.0	0.133	(0.110-0.155)
Subsidized	1,604	58.0	0.198	(0.169-0.227)
**Region of Colombia**				
Amazon	54	1.7	0.160	(0.085-0.234)
Andean	2,316	70.7	0.270	(0.219-0.320)
Caribbean	109	3.3	0.037	(0.023-0.511)
Orinoco	779	23.8	1.809	(1.281-2.338)
Pacific	18	0.5	0.006	(0.002-0.009)

**CI:** confidence interval.

Deaths related to Chagas disease occurred mostly in the Andean region (n = 2,316), the most populated region in Colombia, specifically among people residing in the departments of Santander (n = 754), Bogota (n = 682), Boyaca (n = 349), Norte de Santander (n = 234), and Cundinamarca (n = 158). Deaths in the Orinoco region of Colombia (n = 779,) occurred mainly among people residing in Casanare (n = 384), Meta (n = 239), and Arauca (n = 149). Deaths in most regions of Colombia occurred in people over 65 years of age, except in the Amazon and Caribbean regions, where most deaths occurred in people aged 45-64 years.

We identified chronic forms of Chagas disease in most deaths (98.6%). In contrast, deaths related to the acute form of the disease represented only 1.4%, although these increased by 32.6% in 2000-2018. Death due to acute Chagas disease was most common in the Orinoco region of the country (21.7%).

### Trends in Chagas disease mortality

**FIGURE 1:**
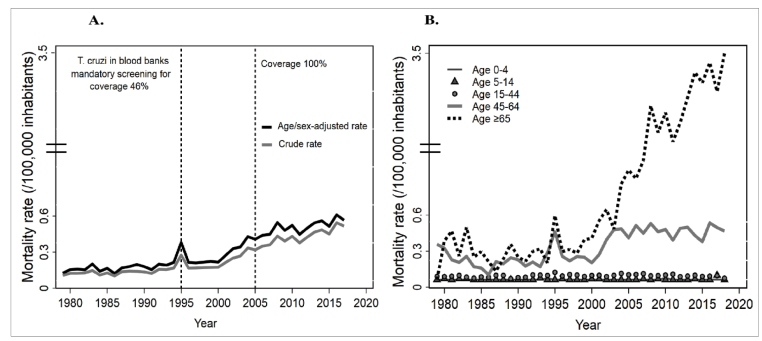
Mortality rates (per 100,000 people) caused by Chagas disease in Colombia, 1979-2018. A) Crude and standardized mortality rates by age, sex, and year of occurrence. B) Standardized mortality rate according to age range and year of occurrence.

In this 40-year period, the age-sex standardized mortality rate had a significant upward trend (APC = 6.60%; 95% CI: 5.9-7.3) with different patterns between the APC of each trend by sex, age group, and geographic region (Figure 1; Table 2). Indeed, the age-sex adjusted mortality rate increased from 0.084 (95% CI: 0.05-0.12) to 0.386 (95% CI: 0.34-0.43) deaths per 100,000 inhabitants in the periods 1979-1989 and 2010-2018, respectively. The highest age- and sex-specific mortality rates were observed in the adult age groups, especially those aged 45-64 years (0.508 deaths per 100,000) and ≥65 years (1.296 deaths per 100,000) (Figure 1). There was a significant increase in age- and sex- adjusted mortality rates in the Orinoco (APC = 8.28%; 95% CI: 6.4-10.2), Caribbean (APC = 5.06%; 95% CI: 3.6-6.5), and Andean (APC = 4.63%; 95% CI: 3.9-5.3) regions, while those in the Pacific region decreased over time (Table 2).

**TABLE 2:** Epidemiological aspects, age- and sex-standardized mortality rates (per 100,000 inhabitants) related to Chagas disease, and annual percentage change in Colombia from 1979 to 2018.

Epidemiological aspects	Mortality rates (per 100,000)		APC*	95% CI	***p* value**
	Initial	Final			
**Sex**					
Female	0.0374	0.2477	5.33	(4.5-6.1)	<0.001
Male	0.0628	0.476	6.00	(4.8-7.2)	<0.001
**Age (years)**					
0-4	0.0018	0.0042	0.19	(−1.1-1.5)	0.742
5-14	0.0019	0.0042	0.18	(−2.2-2.6)	0.815
15-44	0.0498	0.0617	0.88	(−0.3-2.1)	0.118
45-64	0.4321	0.8137	3.61	(2.4-4.8)	<0.001
≥65	0.5860	3.6108	6.72	(5.3-8.2)	<0.001
**Affiliation**					
Contributory	0.0320	0.1463	2.39	(1.9-2.9)	<0.001
Subsidized	0.0511	0.2072	4.88	(4.1-5.7)	<0.001
**Region of Colombia**					
Amazon	0.0042	0.0143	2.91	(1.7-4.2)	<0.001
Andean	0.0367	0.2455	4.63	(3.9-5.3)	<0.001
Caribbean	0.0028	0.0196	5.06	(3.6-6.5)	<0.001
Orinoco	0.0131	0.0836	8.28	(6.4-10.2)	<0.001
Pacific	0.0026	0.0021	−0.42	(−1.1-0.2)	0.223

**APC:** annual percentage change; **CI:** confidence interval.

*Identify changes over time in the linear slope of the trend. Joinpoint regression analysis was used to identify years (as the independent variable) at which significant changes in mortality rate occurred over the study period and the size of these changes.

**FIGURE 2:**
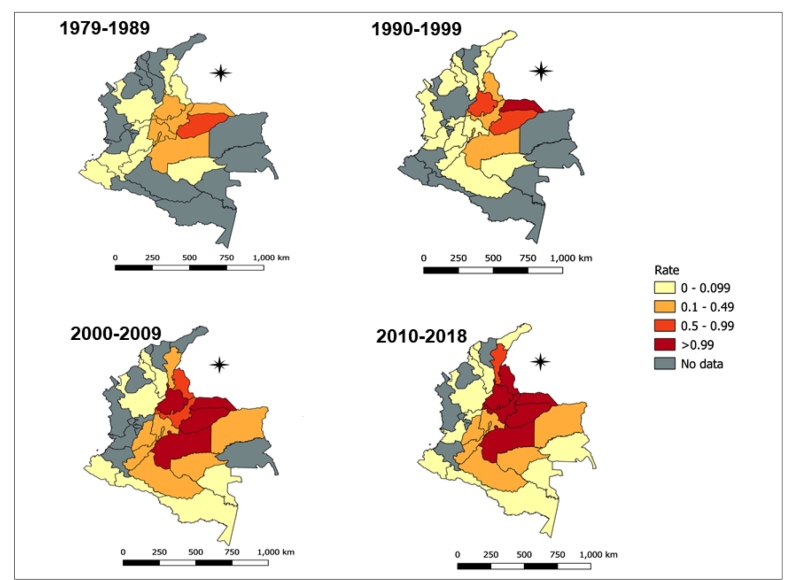
Distribution of mortality rates by Chagas disease (basic cause) according to departments in Colombia from 1979 to 2018. Mortality rates are expressed per 100,000 inhabitants.

The distribution of these death rates was not uniform across the country: the Orinoco region (1.809 deaths per 100,000) had the highest and the Pacific region (0.006 deaths per 100,000) had the lowest age-sex standardized mortality rates due to Chagas disease (Table 1). On the contrary, the departments of Casanare (4.619 deaths per 100,000; 95% CI: 2.96-6.28) and Arauca (2.119 deaths per 100,000; 95% CI: 1.27-2.97), both in the Orinoco region, had higher age-sex standardized mortality rates than the rest of the country. Likewise, the departments of Santander (0.984 deaths per 100,000; 95% CI: 0.72-1.25), Meta (0.918 deaths per 100,000; 95% CI: 0.76-1.07), Boyaca (0.662 deaths per 100,000; 95% CI: 0.47-0.85), Norte de Santander (0.522 deaths per 100,000; 95% CI: 0.36-0.68), and Bogota (0.327 deaths per 100,000; 95% CI: 0.28-0.37) presented age-sex standardized mortality rates above the national average (Figure 2).

## DISCUSSION

The present study highlighted the age- and sex-standardized mortality rates of Chagas disease in Colombia over a 40-year period from 1979 to 2018. The data revealed a general increasing trend in mortality due to Chagas disease at the national level, with different patterns between regions, sex, and age groups. The highest mortality rates were concentrated in the oldest age groups (45-64 years and ≥65 years) and residents in endemic areas, such as the Andean and Orinoco regions.

Consistent with the previous research, our data demonstrated that males and older age groups had an increased risk of death related to Chagas disease^16^ ^-^ ^20^. The predominance of mortality in males may be due to risks acquired through sex-specific roles, certain social and cultural behaviors, and lower utilization of health care services, delaying access to timely diagnosis and treatment^21^ ^,^ ^22^. Although the reason for this is unclear, we have not excluded a biological cause. In contrast, a recent meta-analysis did not find a significant impact of sex on mortality^8^. However, the limited number of included studies and their small sample sizes may have influenced their results.

Our study revealed that the highest mortality due to Chagas disease occurred in the oldest age groups, indicating that the aging population is the most vulnerable to *T. cruzi*. This age-related profile is consistent with recent reports showing that Chagas disease-related mortality rates remain high in infected elderly patients^19^ ^,^ ^23^. Some possible explanations are related to the organization of the healthcare network in different regions of the country and over time^24^. In contrast, this trend could be due to the increase in life expectancy of the population.

These results corroborate the notion that the epidemiology of Chagas disease has changed in recent decades, with a shift towards older age groups^12^. It is important to consider that the longer survival of patients with Chagas disease leads to a higher probability of accumulation of morbidities, especially chronic non-communicable diseases that can increase the demand for health services, worsen the quality of life of people who are also coinfected with *T. cruzi*, and increase mortality^25^ ^-^ ^27^. Undoubtedly, the greater survival of the population with coinfections will represent a difficult challenge for the Colombian health system^5^.

This study displayed that the geographical distribution of the highest mortality rates of Chagas disease is consistent with areas of high endemicity and transmission of vectors reported in previous decades^28^ ^,^ ^29^. The Andean region is the most populated and economically active area in the country, and it presents a great diversity of climate, vegetation, and soils. The main species of domiciled triatomines-*Rhodnius prolixus, Triatoma dimidiata*, and *Triatoma venosa*-have been widely reported in this region^28^. In contrast, the Orinoco region is characterized by extremes of drought and humidity during the year, and its predominant domiciled species include *R. prolixus, T. dimidiata*, and *T. maculata* ^28^. In addition to factors related to endemicity and transmission of vectors, regional differences in mortality of Chagas disease may be related to the migration of rural populations from endemic areas to other regions, reflecting the geographic expansion and urbanization in recent decades, and the regional differences in access to diagnostics and treatment^4^ ^,^ ^12^.

Although only a small percentage of the deaths were caused by acute Chagas disease, these deaths increased in the last study period (2010-2018), especially in the Orinoco region, due to the appearance of new cases of oral infections with *T. cruzi* ^11^. Due to the contamination of food with feces of wild triatomines or with secretions from reservoirs, acute Chagas disease is prevalent in the country^10^. However, the notification of cases in the chronic phase is not mandatory in the entire population but only in pregnant women, those under 18 years of age, indigenous people, and women of childbearing age. 

Additionally, it is striking that Chagas disease is emerging as an opportunistic disease, with reactivation of the disease in people coinfected with HIV^30^ ^,^ ^31^. Although, the reactivation of Chagas disease in cases of acquired immunodeficiency syndrome (AIDS) is not considered a defining condition for severe disease. In contrast to the trend of deaths in older age, deaths from coinfection with HIV predominated in young adults (56.8%), coinciding with studies in Brazil, in which 51.4% of deaths in people coinfected with *T. cruzi* and HIV/AIDS occurred in those aged <50 years of age^31^. *T. cruzi*/HIV coinfection is not recorded in the surveillance system. 

Mortality rates were higher in patients with the subsidized regime, suggesting that the disease persists in populations with low-income socioeconomic conditions. Colombia has made significant progress in health insurance coverage^32^ to the point of almost universal coverage and unification of health benefit plans; this includes diagnosis and treatment for Chagas disease in both regimes. Despite this, the conditions of poverty may be associated with supply barriers that impede the timely access to medical care^4^. Efforts should be directed to guarantee effective access to services and to improve quality.

The trend of increasing mortality rate in this study could be due to the greater coverage of information on mortality in the country as well as a consequence of the creation of the General System of Social Security in Health in 1993. This expanded the coverage of health services, allowing early diagnosis, timely treatment, and adequate clinical follow-up of the disease, to the entire population^33^. Likewise, the control programs for the main vector (*R. prolixus*) and transmission by blood transfusion have favored the increase in the detection and diagnosis of cases, strengthening the notification to the epidemiological surveillance system and the monitoring of deaths^34^. On the contrary, the change from ICD-9 to ICD-10 could influence the mortality rate. ICD-10 generated the most important change since the sixth revision by increasing the number of codes, categories, and large groups of causes of death. In addition, it introduced alphanumeric coding and varied the rules for selecting and modifying the cause of death. A study of correspondences between ICD-10 and ICD-9 due to major causes of death reported that 3.6% of deaths changed groups due to the increase (36.4%) of infectious and parasitic diseases caused by the inclusion of AIDS. This increase in the mortality rate could also be due to changes in epidemiological surveillance actions in this period^35^.

The main limitation of this study is the use of secondary data. It is possible that the number of deaths related to Chagas disease has been underestimated, despite important advances in terms of coverage of the mortality information system as well as in the quality of information on causes of death. On the contrary, for several years, the diagnosis of chronic Chagas disease was centralized in a few laboratories, and we lack information on the detection method for the disease^4^. Despite these limitations, we consider the results of this study to be valid and representative of Colombia. We included multiple causes of death to detect the disease and analyzed age- and sex-standardized analyses.

In conclusion, the mortality analysis conducted in this study indicates that Chagas disease continues to be a public health problem in Colombia with high mortality rates in the older age groups, a wide geographic distribution, considerable regional differences, and an increasing trend. These results highlight the importance of continuing the surveillance and control of Chagas disease transmission using mortality as an additional indicator when planning and evaluating control measures. One of the main challenges is the need to improve clinical care.
